# Metal-deficient SOD1 in amyotrophic lateral sclerosis

**DOI:** 10.1007/s00109-015-1273-3

**Published:** 2015-03-11

**Authors:** James B. Hilton, Anthony R. White, Peter J. Crouch

**Affiliations:** 1 Department of Pathology, The University of Melbourne, Melbourne, Victoria 3010 Australia; 2 Florey Institute of Neuroscience and Mental Health, The University of Melbourne, Melbourne, Victoria 3010 Australia

**Keywords:** Amyotrophic lateral sclerosis (ALS), Motor neuron disease (MND), Copper (Cu), Zinc (Zn), Cu/Zn superoxide dismutase (SOD1), Protein misfolding, Diacetylbis(4-methylthiosemicarbazonato)copper^II^

## Abstract

Mutations to the ubiquitous antioxidant enzyme Cu/Zn superoxide dismutase (SOD1) were the first established genetic cause of the fatal, adult-onset neurodegenerative disease amyotrophic lateral sclerosis (ALS). It is widely accepted that these mutations do not cause ALS via a loss of antioxidant function, but elucidating the alternate toxic gain of function has proven to be elusive. Under physiological conditions, SOD1 binds one copper ion and one zinc ion per monomer to form a highly stable and functional homodimer, but there is now ample evidence to indicate aberrant persistence of SOD1 in an intermediate metal-deficient state may contribute to the protein’s involvement in ALS. This review briefly discusses some of the data to support a role for metal-deficient SOD1 in the development of ALS and some of the outcomes from drug development studies that have aimed to modify the symptoms of ALS by targeting the metal state of SOD1. The implications for the metal state of SOD1 in cases of sporadic ALS that do not involve mutant SOD1 are also discussed.

## Introduction

Amyotrophic lateral sclerosis (ALS) is a fatal neurodegenerative disease that selectively afflicts the motor neurons in the spinal cord, motor cortex and brainstem leading to their dysfunction and eventual death [1]. The loss of functional motor neurons and associated clinical symptoms in ALS is progressive, and despite variability in timeframes relative to clinical subtype, ALS ultimately causes paralysis and premature death which is generally due to respiratory failure. In general, the peak age of onset is 45–60 years, and survival post diagnosis in the vicinity of 3–5 years.

The predominant proportion of ALS cases is characterised as sporadic, with only 5–10 % of cases presenting with a known heritable genetic basis [2]. The most prominent and well-understood genetic basis for familial ALS concerns point mutations to the ubiquitously expressed antioxidant enzyme superoxide dismutase 1 (SOD1) [3]. SOD1 mutations were the first described genetic cause of ALS [4] and are accordingly the most widely studied. They may present as missense mutations or frameshift mutations leading to protein truncation and can reside throughout the protein including within key catalytic and structural regions such as the metal-binding regions and dimer interface [5]. SOD1 mutations are typically segregated into two distinct groups termed the “wild-type-like” mutants (G37R, G93A, A4V, etc.), which retain similar properties to wild-type SOD1, and the metal-binding region mutants (G85R, H46R, etc.), characterised by vastly different biophysical attributes related to metal binding, SOD1 enzymatic activity and spectroscopy [6–9]. But despite gaining good research attention over the past two decades, the definitive mechanisms by which SOD1 mutations cause ALS are yet to be fully elucidated.

## Mutant SOD1 rodent models of ALS

As SOD1 plays a vital role as an antioxidant converting superoxide (O_2_.^−^) into hydrogen peroxide (H_2_O_2_) for subsequent breakdown by glutathione peroxidase and catalase into water [10], it was initially suspected that a loss of function may contribute to ALS pathogenesis. This hypothesis was subsequently rejected since SOD1 knockout mice failed to develop an ALS-like phenotype [11]. These mice did display an increased motor neuron sensitivity to axonal injury [11] and pathology in response to paraquat induced of oxidative stress [12], demonstrating the clear requirement for SOD1 in protecting motor neurons from adverse insults, but they also provided early evidence that a loss of SOD1 function in isolation is not a sole contributor to ALS in mutant SOD1 cases of the disease. Thus, in the absence of an overt phenotype in the SOD1 knockout mice, the main contention is that a toxic gain of function is fundamentally responsible for the development of ALS in cases where SOD1 mutations are involved.

To more thoroughly investigate the role played by the myriad SOD1 mutants discovered in ALS, various rodent models of the disease have been generated by overexpressing human SOD1 containing different point mutations. The initial mutant SOD1 rodent models were developed soon after mutant SOD1 was identified as a cause of ALS in humans, and depending on the form and amount of mutant SOD1 expressed, the rate of disease progression and lifespan of these animals varies considerably [13, 14]. Although the overexpression of mutant human SOD1 in these animals is not an ideal representation of disease conditions in ALS, due to reliance on overexpression of an exogenous gene, these animals nonetheless provide a useful and widely available model for disease investigation. Furthermore, the development of transgenic rodents overexpressing the wild-type form of human SOD1 has provided the opportunity to investigate specific differences between mutant and wild-type SOD1 within the constraints of an overexpression model. Wild-type SOD1-overexpressing mice are generally used as a negative control line due to the absence of an overt ALS-like phenotype in the hemizygous, wild-type SOD1-expressing mice, particularly when compared to age-matched mutant SOD1 expressing mice. However, wild-type SOD1-expressing mice have nonetheless provided some evidence to implicate a role for SOD1 in cases of ALS that do not involve mutant SOD1; subtle phenotype changes have been reported for hemizygous, wild-type SOD1-overexpressing mice at a relatively young age [15], whereas a more recent study has described pronounced ALS-like features in homozygous, wild-type SOD1-overexpressing mice [16].

## SOD1 aggregates

The discovery of insoluble aggregates of SOD1 in mutant SOD1 mouse models of ALS [17] and human cases of ALS [18, 19] led to the postulation that the formation of these aberrant inclusions may represent the toxic gain-of-function mechanism through which SOD1-induced motor neuron death occurs. The production of metal-deficient monomeric species destabilised in a disulphide bond reduced state aided through mutations could present a pathway through which insoluble aggregates can form [20, 21]. Ubiquitin detected within the aggregates [22] suggested that these insoluble inclusions were marked for proteasomal degradation which may have been impeded, perhaps through overloading of the ubiquitin-proteasome system [23]. Moreover, the detection of other proteins such as chaperones within the aggregates [24] indicated the possibility of a secondary loss-of-function disease mechanism through which various proteins become aberrantly sequestered and co-localise within the SOD1 aggregates [25]. The fact that observable insoluble aggregates were typically present at late stages in the disease progression [26], however, suggested that aggregates are likely to be a downstream consequence of alternative pathological processes. With the advent of monoclonal antibodies designed to bind the putative toxic misfolded species of SOD1, attention has shifted towards a soluble form of misfolded SOD1 which was reported to be detectable across all ages in mutant SOD1 expressing murine spinal cord tissue [27]. Supporting this finding, it was observed that co-expression of mutant and wild-type human SOD1 increased the solubility of mutant misfolded SOD1 species leading to greater cellular toxicity [28], demonstrating that the soluble form may be an earlier contributor to ALS pathogenesis whilst aggregate formation may represent a cellular defence mechanism [28].

## Zinc-deficient SOD1

Once translated, the incipient monomeric polypeptide of SOD1 becomes bound to one zinc atom providing structural integrity before the direct interaction with copper chaperone for superoxide dismutase (CCS) which ensures the binding of a copper atom required for catalytic activity [29]. The establishment of a fully functional SOD1 enzyme then occurs through intramolecular disulphide bond formation and combination with another monomeric protein to create the functional homodimer [30]. The fact that many disparate point mutations within SOD1 can induce ALS led to the hypothesis that an impaired folding mechanism may be responsible for producing aberrant copper-mediated chemistry. Changes in SOD1 protein folding due to mutations may allow aberrant substrate access to the copper-dependent catalytic region leading to potentially harmful reactions. Furthermore, impairment in copper-binding capacity may promote its release where it could catalyse reactions that cause oxidative damage [31]. An initial substrate proposed to induce cellular pathology was peroxynitrite [32] which is created spontaneously through interaction between nitric oxide (NO) and O_2_.^−^, leading to tyrosine nitration of proteins capable of interfering with cellular pathways. However, the fact that the G85R SOD1 mutation causes rapidly progressing disease without retaining copper-dependent enzymatic activity [33, 34], in conjunction with studies showing co-expression of wild-type SOD1—which would be expected to ameliorate peroxynitrite damage—worsens disease progression [35], have made this a less likely hypothesis. The ability for H_2_O_2_ to induce peroxidation damage on cellular components and its central position within the antioxidant role of SOD1 provided another popular candidate for the SOD1-dependent toxic species. But, the same limitations apply as for the peroxynitrite hypothesis where SOD1G85R mutations induce an ALS phenotype independent of aberrant copper chemistry, with further evidence demonstrating an inability to detect elevated levels of H_2_O_2_ in mutant SOD1 models [36].

**Fig. 1:**
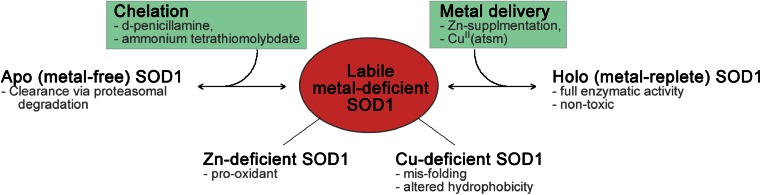
Overview of various forms of SOD1 relative to metal state, the potential contribution of metal-deficient SOD1 to ALS and therapeutic opportunity to attenuate toxicity of metal-deficient SOD1

Zinc is crucial for the structural integrity of SOD1 and possesses a curiously weaker affinity than copper [37], prompting the suggestion that a zinc-deficient species of SOD1 may contribute to ALS (Fig. 1). It has been reported that mutant forms of SOD1 have a decreased affinity for the structural zinc atom in contrast to the wild-type species [38], presumably affecting the protein folding pattern and indirectly affecting the catalytic activities of SOD1. Changes to the native zinc binding site lead to increased zinc loss from SOD1 which subsequently becomes more relaxed and potentially more accessible to substrates able to catalyse nitration reactions. Additionally, through a shared bridging histidine residue [39], the consequence of zinc deficiency may be to change the redox state of the bound copper which could facilitate protein nitration [37]. Neurofilaments and metallothioneins bind readily to metals [40, 41] and are present in large cellular concentrations similar to SOD1, so it has been proposed that these proteins may act as a sink for zinc, leading to greater zinc-deficient SOD1 levels and therefore increased peroxynitrite-dependent nitration reactions [37, 42]. Due to reported similarities in the binding affinity for zinc between mutant SOD1 and the wild-type form, it has been contended that there may exist the capacity for wild-type SOD1 to become zinc deficient in sporadic cases of ALS as well [38, 43]. More recently, it was reported that zinc-deficient SOD1 is required to induce motor neuron death with copper chelation acting to protect motor neurons from nitric oxide-induced death and the addition of fully metallated wild-type SOD1 acting to increase toxicity in the presence of NO; it was demonstrated that the fully metallated monomer of wild-type SOD1 could form a dimer with a zinc-deficient monomer of mutant SOD1, thereby stabilising the zinc-deficient monomeric species and exacerbating its toxicity in the presence of NO [44].

Initial studies on zinc supplementation as a treatment for mutant SOD1 mice demonstrated an increase in the rate of death [45]. A follow-up study however attributed this outcome to excessive zinc treatment leading to competition for copper absorption that ultimately inhibited ceruloplasmin resulting in fatal anaemia [46]. An amended treatment programme using a more modest zinc supplement concentration conversely demonstrated an improved survival rate in the same mouse model of ALS [46]. Whilst the zinc-deficient SOD1 induction of peroxynitrite-dependent pathology is widely supported, contradictory evidence exists for the relevance of tyrosine nitration in most SOD1 mutant models as well as human ALS cases [36, 47]. Considering the requirement of NO for peroxynitrite production, the abatement of nitric oxide synthase (NOS) generators of NO would be expected to ameliorate disease symptoms. Yet the pharmacological inhibition of neuronal NOS [48] and genetic ablation of inducible NOS [49] failed to have an effect on the survival of SOD1 mutant mice, casting some doubt upon the peroxynitrite hypothesis.

## Copper-deficient SOD1

As an alternative to zinc-deficient SOD1 driving toxicity in specific models of familial ALS, a role for a copper-deficient species has been mooted (Fig. 1). SOD1 has one of the greatest affinities for copper, alongside metallothioneins, with a reported ∼7000 times greater affinity for copper than zinc in the case of wild-type SOD1 that was increased further in A4V SOD1 mutants [43]. As previously mentioned, the SOD1 loss-of-function hypothesis was rejected based on an absence of ALS-like pathology in SOD1 knockout mice, demonstrating that a loss of copper-dependent dismutase activity in isolation does not cause ALS [12]. Additionally, when endogenous mouse SOD1 was knocked out in mice expressing the metal-binding region SOD1G85R mutant—which exhibits extremely poor metal-binding properties [50]—there was no difference in survival or age of symptom onset [51]. Work performed on the wild-type-like SOD1G37R mutant described above conversely demonstrated dismutase activity levels that were unchanged relative to wild-type SOD1, yet induced an ALS-like phenotype when expressed in transgenic mice [52]. Subsequent work investigating the metallation status of mutant SOD1 species found that whilst metal-binding region SOD1 mutants were severely metal deficient, wild-type-like mutant SOD1 species exhibited normal activity levels per equivalent copper but a detectable decrease in metal binding capacities relative to wild-type control [7]. This was supported through computational analysis performed on the SOD1G37R mutant which showed that this point mutation, which is not within the metal binding region, could also induce metal binding impairment leading to decreased copper affinity ahead of zinc based on free energy calculations [53].

Several studies, utilising copper chelating compounds in SOD1 mutant models of ALS intended to inhibit the purportedly aberrant copper chemistry, have reported protection against motor neuron loss with a corresponding improvement in lifespan and locomotor function [54–56]. The mechanisms of action for therapeutic benefit suggested attenuation of copper ion toxicity [55], decreased spinal cord copper ion levels and reduced lipid peroxidation [56], or decreased markers of oxidative damage and inflammation [54]. Contrarily, previous work on the successful imaging compound diacetylbis(4-methylthiosemicarbazonato)copper^II^ [57] demonstrated that under conditions of impaired mitochondrial transport chain function and elevated NADH levels, treatment with Cu^II^(atsm) increased intracellular retention of copper [58]. When used to treat a SOD1G93A mouse model of ALS, the compound significantly delayed locomotor deficit onset and improved survival, decreased levels of peroxynitrite-induced protein nitration and increased SOD1 activity in spinal cord tissue [57]. Collectively, these studies indicated that a therapeutic agent capable of increasing copper bioavailability could protect against mutant SOD1 toxicity, a possibility consistent with previous in vitro studies which had described the potential for copper deficiency to promote SOD1 pathology via increased misfolding and altered hydrophobicity [59, 60]. It was however a more recent study [61] involving Cu^II^(atsm) treatment in a SOD1G37R mouse model of ALS that illustrated the potential link between copper-deficient SOD1 and ALS pathogenesis in vivo. Oral treatment with the compound again showed improved locomotor function and prolonged survival compared to untreated mice, concomitant with decreased motor neuron death within the spinal cords of treated mice. Using a FTICR mass spectrometry technique [62, 63], it was shown that zinc-deficient mutant SOD1 levels in both treated and untreated mutant mice were relatively low (∼1 μM), in contrast to the substantially more abundant (∼60 μM) copper-deficient form of the protein. Treatment with Cu^II^(atsm) induced a significant decrease in the levels of metal-deficient mutant SOD1 and a corresponding increase in fully metallated holo form of the protein. Changes to the metal-deficient pool correlated directly to changes in the copper-deficient pool. It was proposed that the Cu^II^(atsm) treatment delivered copper directly to the mutant SOD1, and this was supported by an experiment in which the mice were treated with isotopically labelled ^65^Cu^II^(atsm). Due to conversion of the metal-deficient SOD1 to the highly stable holo form, total levels of mutant SOD1 were increased in the Cu^II^(atsm)-treated mice. Based on the fact that the phenotype of these mice was improved by the Cu^II^(atsm) treatment, despite an overall increase in levels of mutant SOD1, it was concluded that the metal state of the SOD1 is a greater determinant of the protein’s role in motor neuron death and the ALS-like phenotype of these animals than the mutant amino acid sequence per se. Interestingly, the overall increase in SOD1 levels in the Cu^II^(atsm)-treated mice also included an increase in levels of the protein detected using antibodies selective for the misfolded species [61].

Taken together, these results indicate that improving the copper bioavailability within the spinal cord tissue of SOD1 mutant mice and improving SOD1 metallation status are potential mechanisms for attenuating the ALS phenotype. Furthermore, since the unmetallated apo form of SOD1 is rapidly turned over [64] and the fully metallated form is stable and likely to be non-toxic regardless of amino acid substitution mutations [65–68], it appears that a partially metal-deficient intermediate species may be responsible for the SOD1 toxic gain of function. This could potentially explain the contradictory data in the literature reporting therapeutic benefits from both copper chelating compounds and “copper delivery agents”, both of which may be acting to shift the equilibrium away from this potentially toxic copper-deficient species of SOD1 (Fig. 1). It is currently not clear why such a large pool of copper-deficient SOD1 would accumulate within the spinal cord tissue of SOD1G37R mice, but outcomes from the SOD1G93A mouse model have demonstrated that widespread and progressive impairment of intracellular copper trafficking occurs due to expression of the mutant SOD1 and that these changes to copper trafficking can be detected at a presymptomatic stage [69]. Thus, it is apparent that copper homeostasis is substantially altered by the expression of mutant SOD1 and that therapeutically modulating copper bioavailability can attenuate ALS-like symptoms in mutant SOD1 mice.

Definitive mechanistic data to support altered copper homeostasis as a valid therapeutic basis for treating ALS in the clinic, particularly sporadic cases of the disease, are yet to be reported. However, mutations to the copper transporter ATP7A that lead to systemic copper deficiency are an established cause of the infantile-onset neurodegenerative Menkes disease [70, 71], and some ATP7A missense mutations have been shown to cause X-linked distal hereditary motor neuropathies in the absence of systemic copper deficiency [72]. This suggests that motor neurons may be particularly sensitive to altered copper homeostasis, and this has been supported recently by a report describing a degenerative phenotype (involving progressive muscular atrophy, loss of motor neuron cell bodies and denervation of the neuromuscular junction) in mice caused by selective knockout of the *Atp7a *gene from motor neurons [73]. Although such an explicit role for altered copper homeostasis as a cause of ALS is not yet evident, these studies nonetheless indicate the fundamental requirement for copper in maintaining motor neuron functionality.

## Concluding remarks

After more than 20 years of research since SOD1 mutations were discovered as a definitive cause of ALS, the mechanisms by which SOD1 cause ALS remain far from elucidated. Transgenic SOD1 animal models that develop robust ALS-like phenotypes have been created and have been used extensively for preclinical drug development, but instead of providing clear insight to the pathogenesis of ALS, these animal models have often been the source of apparent confounding outcomes. But progress in being made and the role for the metal state of SOD1 in ALS is one area of research providing new opportunity for the development of effective therapeutic options. It is becoming clear that the metal state of SOD1 can be modulated via therapeutic intervention and that the metal state of SOD1 may be a greater determinant of the protein’s elusive toxic gain of function than the disease-causing mutations themselves. Further to this, evidence for a toxic gain of function and structural instability in metal-deficient wild-type SOD1 indicates a mechanism by which SOD1 contributes to cases of ALS that do not involve SOD1 mutations. Whether or not metal-deficient wild-type SOD1 contributes to the pathogenesis of sporadic ALS is yet to be established, but if proven could provide opportunity to treat all cases of ALS, not just mutant SOD1 cases, using drugs designed to improve the protein’s metal state.
